# Xylosylation of the Notch receptor preserves the balance between its activation by *trans*-Delta and inhibition by *cis*-ligands in *Drosophila*

**DOI:** 10.1371/journal.pgen.1006723

**Published:** 2017-04-10

**Authors:** Tom V. Lee, Ashutosh Pandey, Hamed Jafar-Nejad

**Affiliations:** 1 Department of Molecular and Human Genetics, Baylor College of Medicine, Houston, Texas, United States of America; 2 Program in Developmental Biology, Baylor College of Medicine, Houston, Texas, United States of America; Harvard Medical School, Howard Hughes Medical Institute, UNITED STATES

## Abstract

The *Drosophila* glucoside xylosyltransferase Shams xylosylates Notch and inhibits Notch signaling in specific contexts including wing vein development. However, the molecular mechanisms underlying context-specificity of the *shams* phenotype is not known. Considering the role of Delta-Notch signaling in wing vein formation, we hypothesized that Shams might affect Delta-mediated Notch signaling in *Drosophila*. Using genetic interaction studies, we find that altering the gene dosage of *Delta* affects the wing vein and head bristle phenotypes caused by loss of Shams or by mutations in the Notch xylosylation sites. Clonal analysis suggests that loss of *shams* promotes Delta-mediated Notch activation. Further, Notch *trans*-activation by ectopically overexpressed Delta shows a dramatic increase upon loss of *shams*. In agreement with the above *in vivo* observations, cell aggregation and ligand-receptor binding assays show that *shams* knock-down in Notch-expressing cells enhances the binding between Notch and *trans*-Delta without affecting the binding between Notch and *trans*-Serrate and cell surface levels of Notch. Loss of Shams does not impair the *cis*-inhibition of Notch by ectopic overexpression of ligands *in vivo* or the interaction of Notch and *cis*-ligands in S2 cells. Nevertheless, removing one copy of endogenous ligands mimics the effects of loss *shams* on Notch *trans*-activation by ectopic Delta. This favors the notion that *trans*-activation of Notch by Delta overcomes the *cis*-inhibition of Notch by endogenous ligands upon loss of *shams*. Taken together, our data suggest that xylosylation selectively impedes the binding of Notch with *trans*-Delta without affecting its binding with *cis*-ligands and thereby assists in determining the balance of Notch receptor’s response to *cis-*ligands vs. *trans-*Delta during *Drosophila* development.

## Author summary

One of the key mechanisms used by neighboring cells to communicate with each other in animals is signaling through an evolutionarily conserved receptor family called Notch. Binding of Notch to ligands of the Delta/DLL and Serrate/JAG families from neighboring cells leads to activation of the signaling pathway and is called *trans*-activation. In contrast, interaction of Notch with ligands expressed in the same cell has an inhibitory effect on signaling and is known as *cis*-inhibition. The balance between *trans*- and *cis*- interactions ensures optimum Notch signaling in some contexts during animal development. We have used the model organism *Drosophila* (fruit fly) to decipher the mechanism through which a carbohydrate residue, xylose, regulates Notch signaling in specific contexts. We provide evidence that addition of xylose residues to the Notch receptor decreases its interaction with *trans*-Delta ligand without affecting its interaction with *cis*-ligands. Thereby, xylose tunes the Notch pathway by modulating the balance between Notch *trans*-activation by Delta and Notch *cis*-inhibition by same-cell ligands. Misregulation of Notch signaling causes a number of human diseases including cancer and developmental disorders. Therefore, understanding the role of xylosylation in Notch signaling can potentially establish a new framework for therapeutic targeting of this pathway.

## Introduction

Notch signaling is a juxtacrine signaling pathway broadly used during animal development and tissue homeostasis [1]. Both Notch and its ligands are type I transmembrane proteins containing multiple epidermal growth factor-like (EGF) repeats, which are involved in Notch-ligand interactions and are the sites of several *O*-linked sugar modifications [2,3]. Interaction of the Delta/Serrate/Lag-2 (DSL) ligands on the surface of the signal-sending cell and the Notch receptor on the signal-receiving cell initiates Notch *trans*-activation and results in the induction of Notch downstream targets [4,5]. In contrast, binding of Notch and ligands expressed in the same cell can result in *cis*-inhibition of the pathway [6–10]. The balance between the *cis* and *trans* interactions of Notch with ligands is thought to determine whether each cell assumes a signal-sending or a signal-receiving role with regard to a given ligand [11,12].

The Notch ligands Delta and Serrate function redundantly in several contexts during fly development [13]. However, there are developmental processes in which Delta and Serrate show non-redundant roles [14–17]. For example, although Serrate plays a minor, fully redundant role during wing vein formation, Delta is the ligand primarily involved in wing vein development [13,15,18]. In this context, both *trans*-activatory and *cis*-inhibitory interactions between Delta and Notch are important for demarcation of the boundary between vein and intervein tissues [15,18]. Specifically, high levels of Delta in the central provein cells are thought to *cis*-inhibit Notch and allow the cells to assume the wing vein fate. Delta from central provein cells also *trans*-activates Notch in neighboring cells to prevent the adoption of vein fate and to establish the wing-intervein boundary. The haploinsufficient wing vein phenotypes exhibited by *Notch*^*+/–*^and *Delta*^*+/–*^animals and their mutual suppression in *Notch*^*+/–*^; *Delta*^*+/–*^double-heterozygous flies [19] further indicate that both *cis*-inhibition and *trans*-activation of Notch by Delta are required for proper wing vein formation, and that altering the relative expression levels of these two proteins can tip the balance between *cis*- and *trans*-interactions between them. However, it remains to be determined whether other mechanisms exist to regulate this balance.

Sugar modifications of the Notch receptors are known to affect Notch pathway activation at various steps, including folding, trafficking, ligand binding and potentially cleavage [20–22]. For instance, addition of *N*-acetylglucosamine (GlcNAc) to *O*-fucose residues on Notch EGF repeats by Fringe proteins differentially regulates the response of Notch to its ligands by regulating Notch-ligand interactions [23–26]. It is noteworthy that Fringe proteins promote the binding of Notch receptors to Delta ligands both in *cis* and in *trans* configurations [11] and as such, are not likely to be involved in regulating the balance between these opposing activities of Delta ligands. Another type of Notch sugar modification is the addition of *O-*glucose onto Notch EGF repeats by the protein *O*-glucosyltransferase 1 (Poglut1). In *Drosophila*, Poglut1 is encoded by *rumi* [27] and promotes Notch activation [27–30]. *O-*glucosylated EGF repeats also serve as a substrate for the addition of xylose by glucoside xylosyltransferase (GXYLT) enzymes [31,32]. Functional studies of the fly GXYLT, Shams, indicate that addition of xylose onto a specific subset of Notch EGF repeats (EGF16-20) negatively regulates Notch signaling in specific contexts, i.e. wing veins and head bristles [32]. Loss of xylose from Notch results in increased cell surface expression of Notch in the pupal wing but not in third instar larval wing discs [32], suggesting that additional mechanisms underlie the context-specificity of the *shams* loss-of-function phenotype.

Here, we provide evidence that Notch xylosylation by Shams decreases Delta-mediated *trans*-activation of Notch by reducing the binding of Notch to *trans*-Delta without affecting the binding of Notch to *cis*-ligands. The effect of loss of Shams on *trans*-activation of Notch by overexpressed Delta can be mimicked by decreasing the level of endogenous *cis-*ligands, suggesting that upon loss of Notch xylosylation, Delta *trans*-activation overcomes Notch *cis*-inhibition by ligands. Altogether, our observations indicate that Shams regulates the balance between *trans*-activation and *cis*-inhibition of Notch by Delta to ensure optimal Notch activation in several contexts during *Drosophila* development.

## Results

### Increased gene dosage of *Delta* enhances the wing vein loss upon lack of Notch xylosylation

**Fig 1:**
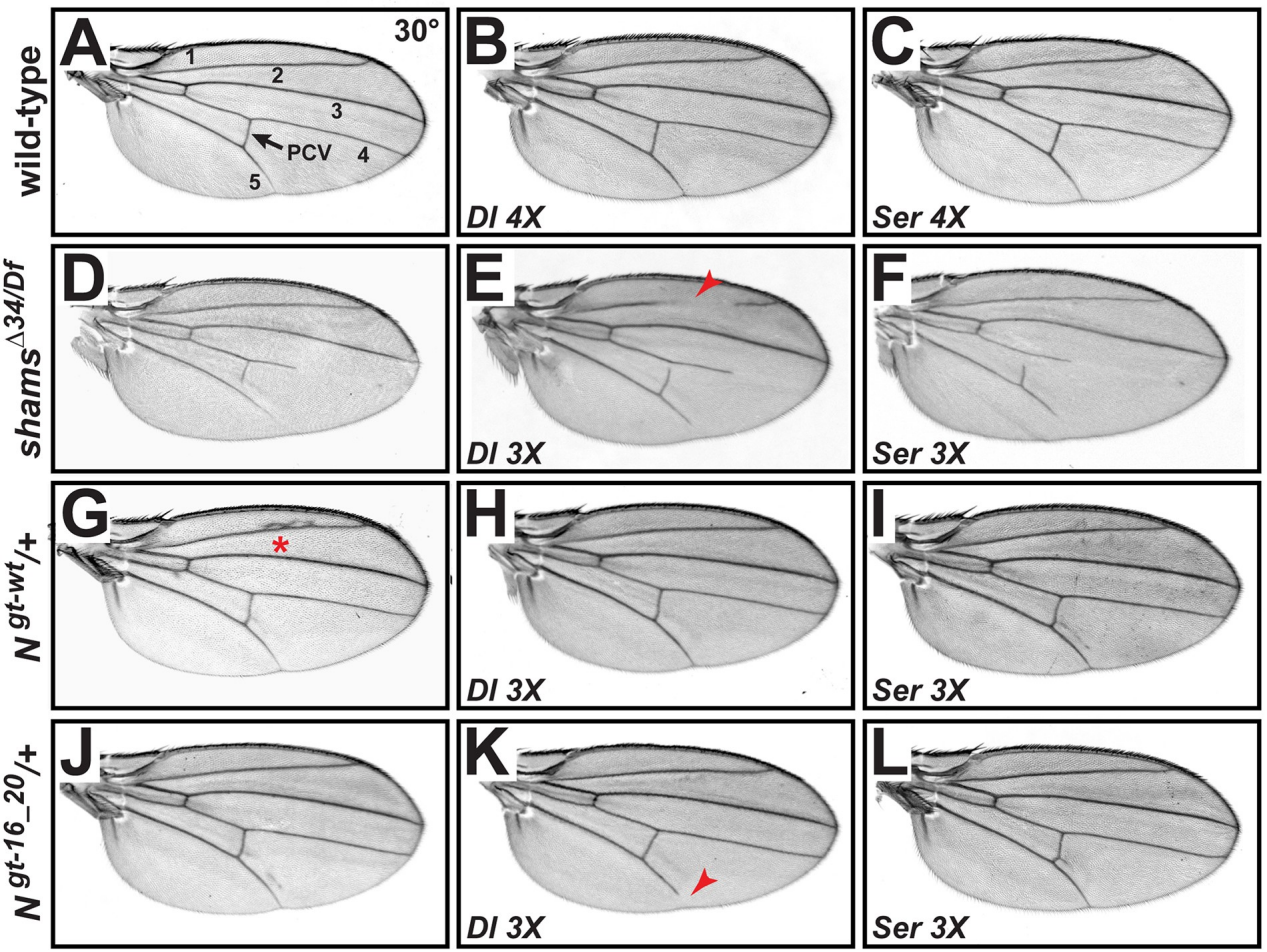
Shams inhibits Notch activation in response to increased levels of Delta but not Serrate. All wings are from females raised at 30°C. (A) Adult wing of a wild-type fly. Wing veins are numbered. The arrow marks the posterior cross-vein (PCV). (B,C) Adding two additional genomic copies of *Delta* (*Dl*^*gt-wt*^) (B) or *Serrate* (*Ser*^*gt-wt*^) (C) does not affect adult wings. (D) *shams*^*Δ34/Df*^ mutants partially lose L4 and L5 wing veins. (E) *Dl*^*gt-wt/+*^ *shams*^*Δ34/Df*^ animals have a partial loss of L2 vein (red arrow), which is not observed in *shams*^*Δ34/Df*^ animals. (F) *Ser*^*gt-wt/+*^ *shams*^*Δ34/Df*^ wings are indistinguishable from *shams*^*Δ34/Df*^ wings (compare to D). (G) Providing an additional genomic copy of *Notch* (*N*^*gt-wt*^) induces extra wing vein material at the L2 (red asterisk) and occasionally at the L5 wing vein indicative of Confluens phenotype. (H) *N*^*gt-wt/+*^; *Dl*^*gt-wt/+*^ animals exhibit no wing vein defects (compare to G). (I) *N*^*gt-wt/+*^; *Ser*^*gt-wt/+*^animals do not show extra vein around L2 but occasionally show extra wing vein near L5. (J) Providing an additional genomic copy of *Notch* lacking the functional xylosylation sites (*N*^*gt-16_20*^) induces extra wing vein material near the L5 wing vein. (K) *N*^*gt-16_20/+*^; *Dl*^*gt-wt/+*^animals exhibit a partial loss of L5 (71% penetrant, n = 17). (L) *N*^*gt-16_20/+*^; *Ser*^*gt-wt/+*^ wings are indistinguishable from *N*^*gt-16_20*^ wings (n = 15) (compare to J).

To assess the role of Delta in the wing vein loss phenotype observed in *shams* mutants, we performed gene-dosage experiments using *Delta* genomic rescue transgenes [11]. Providing two additional genomic copies (4X) of *Delta* in a wild-type background does not generate any adult wing phenotypes at 30°C (Fig 1A and 1B) or at room temperature [11]. The absence of phenotype is likely due to a simultaneous increase in the level of *cis*-inhibition and *trans*-activation of Notch upon increasing ligand levels. As previously reported, loss of *shams* results in a temperature-sensitive loss of distal part of adult wing veins L4 and L5 and a partial loss of the posterior cross-vein (Fig 1D) [32]. In a *shams*^*Δ34/Df*^ null background, providing one additional copy of *Delta* results in a fully penetrant, partial loss of wing vein L2 in addition to L4, L5 and posterior cross vein (Fig 1E), suggesting that *shams* mutants are sensitive to increased Delta levels compared to control animals. We performed similar genetic interaction experiments in flies harboring wild-type *Notch*^*gt-wt*^ or xylosylation-deficient *Notch*^*gt-16_20*^ genomic transgenes [28]. Increasing the gene dosage of *Delta* does not result in wing vein loss in *N*^*+/+*^; *N*^*gt-wt*^*/+* animals, which have three copies of the wild-type *Notch* (Fig 1G and 1H). However, providing an additional copy of *Delta* in *N*^*+/+*^; *N*^*gt-16_20*^*/+* animals results in a partially penetrant loss of the distal wing vein L5 (Fig 1J and 1K), which resembles the *shams* mutant phenotype at 25°C [32]. Together, these data indicate that Notch signaling in *shams* mutants is sensitive to Delta levels and support the hypothesis that lack of Notch xylosylation affects Delta-mediated signaling. We also examined the effects of a *Serrate* transgene in similar experiments. Providing two additional copies of *Serrate* does not generate any wing vein loss in a wild-type background (Fig 1C) [11]. Moreover, increasing *Serrate* gene dosage does not enhance the wing vein loss phenotype in a *shams*^*Δ34/Df*^ null background (Fig 1F). Finally, *N*^*+/+*^; *N*^*gt-wt*^*/+* and *N*^*+/+*^; *N*^*gt-16_20*^*/+* animals do not exhibit wing vein loss upon addition of an extra copy of *Serrate* (Fig 1I and 1L). These results indicate that in the context of wing vein formation, lack of xylosylation does not render Notch sensitive to Serrate levels.

### Removing one copy of *Delta* suppresses the loss of wing vein and head bristles in *shams* mutants

**Fig 2:**
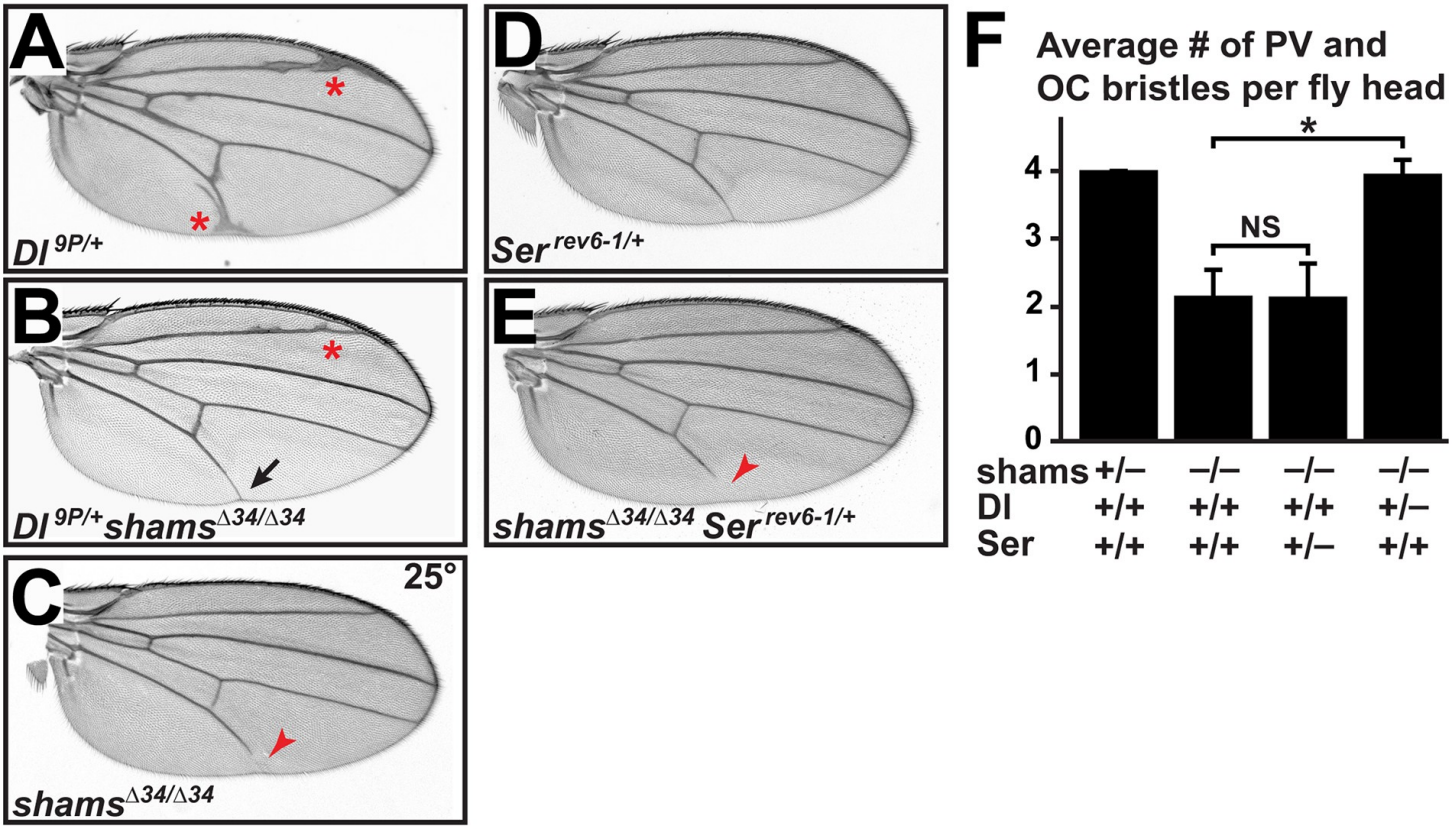
Removing one copy of *Delta* suppresses the loss of wing vein and head bristles in *shams* mutants. (A-F) All animals raised at 25°C. (A) Adult wing from a *Dl*^*9P/+*^ animal. Note the wing vein thickening and extra wing vein material (red asterisks). (B) *Dl*^*9P/+*^ *shams*^*Δ34/Δ34*^ adult wings show a suppression of the vein thickening and extra wing veins present in *Dl*^*9P/+*^ mutants (compare to A) and the wing vein loss (black arrow) present in *shams*^*Δ34/ Δ34*^ mutants (red arrowhead in C). (D) *Ser*^*rev6-1/+*^ adult wings do not exhibit any defects. (E) *shams*^*Δ34/Δ34*^ *Ser*^*rev6-1/+*^animals exhibit wing vein loss similar to *shams*^*Δ34/Δ34*^ mutants (compare to C). (F) Shown is the average number of post-vertical (PV) and ocellar (OC) bristles in adult heads of the indicated genotypes. *shams*^*Δ34/+*^ animals have a total of 4 PV and OC bristles, similar to wild-type animals. Removing one copy of *Delta* but not *Serrate* restores the head bristles in *shams* mutant animals. Error bars are standard error of mean. *P<0.001, NS: not significant.

Genetic interaction experiments were performed to examine the effect of decreasing Delta levels on the *shams* mutant phenotypes. Loss of one copy of *Delta* in *Delta*^*9P/+*^ (*Dl*^*9P/+*^) animals results is extra wing vein material (Fig 2A) [33]. When one copy of *Delta* is removed in a *shams*^*Δ34/Δ34*^ background, the *shams* mutant wing vein loss is completely suppressed, and the extra wing vein phenotype of *Dl*^*9P/+*^ is partially suppressed (Fig 2B and 2C). We have previously reported that loss of *shams* also results in the loss of post-vertical (PV) and ocellar (OC) bristles in the adult head [32]. Genetic interaction studies indicate that removing one copy of *Delta* in *shams* mutants rescues the loss of head bristles (Fig 2F) similar to the wing vein loss phenotype. Together, these observations support the notion that the *shams* loss-of-function phenotypes are due to increased Delta-mediated signaling. We also examined the effect of decreasing Serrate levels on the above-mentioned phenotypes (loss of wing vein and head bristles). Removing one copy of *Serrate* does not affect the loss of wing vein and head bristles in *shams* mutants (Fig 2D–2F). These observations indicate that altered Serrate-mediated signaling is not likely to contribute to *shams* loss-of-function phenotypes. Surprisingly, removing one copy of *Serrate* in *shams*^*Δ34/Δ34*^ mutant animals results in wing margin loss in some animals (S1A Fig; 21% penetrant, n = 73), which resembles the loss-of-function mutants of *Notch* and *Serrate*. Adding one copy of a *Serrate* genomic rescue transgene [11] fully rescues the wing margin phenotype without affecting the wing vein loss caused by the loss of *shams* (S1B Fig), indicating that the observed wing margin loss is indeed due to decreased Serrate levels in a *shams* mutant background. This indicates that in the wing margin, *shams* might play a redundant role which is revealed when one copy of *Serrate* is removed. Thus, a role for Shams in Serrate-mediated Notch signaling remains a distinct possibility in the context of wing margin formation.

### Loss of *shams* enhances Delta-mediated Notch activation in *Drosophila* wing tissue

**Fig 3:**
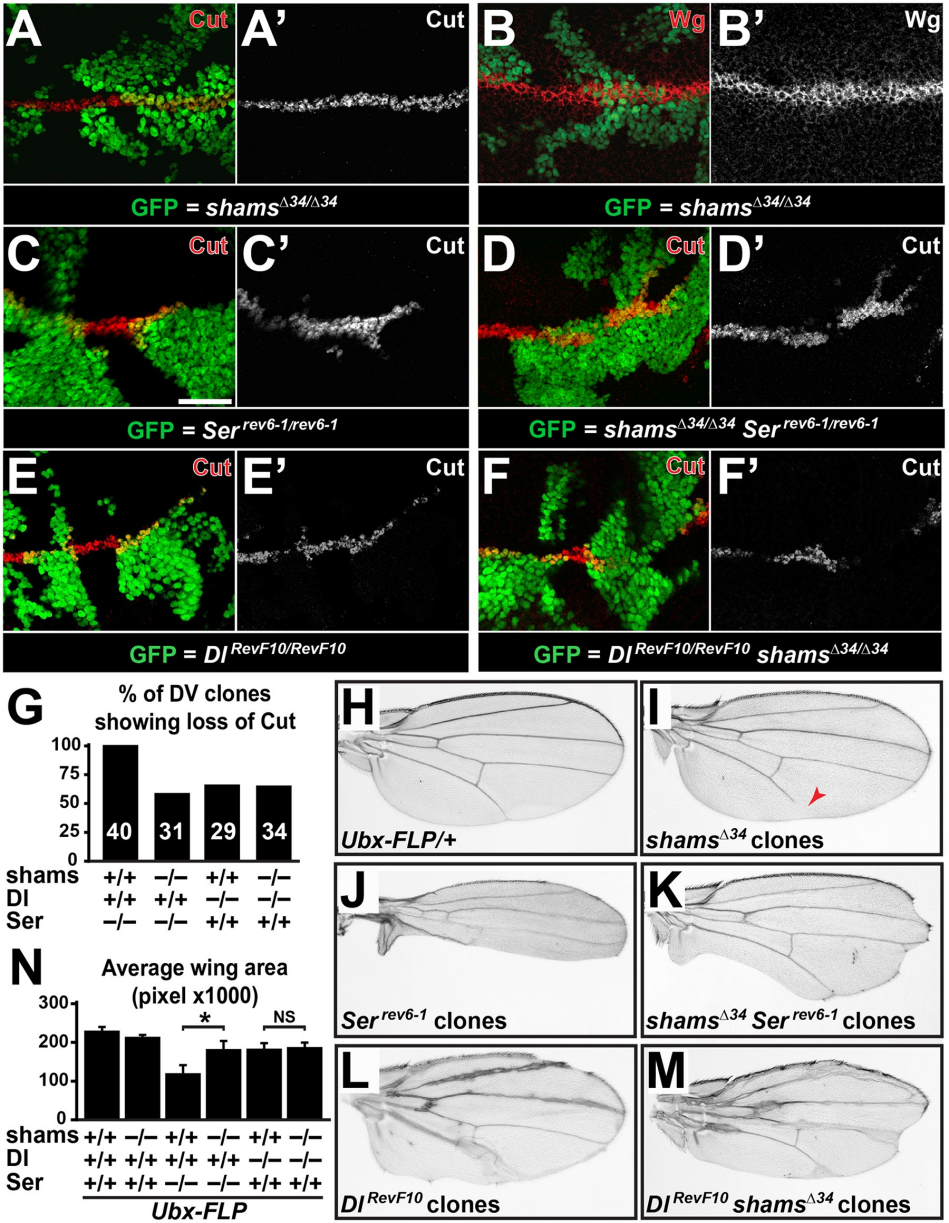
Simultaneous loss of *shams* partially rescues Notch signaling in *Serrate* clones spanning the DV boundary, where Delta is the only ligand. (A-N) All animals raised at 25°C. (A-F’) Third instar wing imaginal discs with mutant cells marked by the presence of GFP. Scale bar in (C) is 25 μm and applies to A-F’. Dorsal is up, anterior to the left. (A-B’) *shams*^*Δ34*^ mutant clones do not seem to show altered Cut (A-A’) and Wingless (Wg) (B,B’) expression domains at the developing wing margin except a slight broadening in clone area compared to wild-type. (C,C’) *Ser*^*rev6-1*^ mutant clones lack Cut expression (red) except for cells directly adjacent to wild-type cells (number of clones n = 40). (D,D’) *shams*^*Δ34*^ *Ser*^*rev6-1*^ double-mutant clones often express Cut in cells at the clonal boundary and within the mutant clones (number of clones n = 31). (E-F’) Comparable lack of Cut expression in *Dl*^*RevF10*^ mutant clones (E,E’; number of clones n = 29) and *Dl*^*RevF10*^ *shams*^*Δ34*^ double mutant clones (F,F’; number of clone n = 34). (G) Graph represents % of mutant clones crossing the DV boundary which show loss of Cut expression in indicated genotypes. Number of clones scored for each genotype is shown. (H-M) Adult wings harboring the indicated single or double-mutant clones generated by the *Ubx-FLP* transgene. Clones are not marked. (N) Graph represents average wing area (n = 13 for each genotype) in the indicated genotypes. Error bars are standard error of mean. *P<0.05, NS: not significant.

To examine the effects of loss of *shams* on the Notch activity in the wing imaginal disc, we performed clonal analysis in the 3rd instar larval wing discs by using the MARCM system [34]. We expressed Flippase with an Ultrabithorax enhancer (*Ubx-FLP*), which induces FRT-mediated recombination in the wing imaginal discs with a high efficiency [27,35]. We first examined the effect of loss of *shams* on Notch activity by staining for Notch targets Cut and Wingless (Wg) in *shams*^*Δ34*^ mutants clones. These mutant clones do not exhibit clear abnormalities in the expression of Cut and Wg when compared to adjacent wild-type or heterozygous cells (Fig 3A–3B’). However, slight broadening observed in Cut and Wg expression in *shams*^*Δ34*^ mutants clones supports a mild increase in Notch activation (Fig 3A–3B’). To further assess the effect of loss of *shams* on ligand-mediated Notch activity, we examined Cut expression in single-mutant clones for ligands (*Ser*^*rev6-1*^ and *Dl*^*RevF10*^) and double-mutant clones for ligands and *shams* (*shams*^*Δ34*^ *Ser*^*rev6-1*^ and *Dl*^*RevF10*^ *shams*^*Δ34*^). To analyze the effects of loss of *shams* on each ligand without interference from the other ligand, we focused on mutant clones which cross the dorsal-ventral (DV) boundary. *Ser*^*rev6-1*^ clones show a highly penetrant loss of Cut, except for the cells that directly abut the wild-type tissue (Fig 3C, 3C’ and 3G; n = 40). This observation indicates that when cells around the DV boundary lack *Serrate*, they fail to activate the Notch target Cut despite the presence of *Delta* in the clones. In contrast, Cut-positive cells non-adjacent to the clone boundary are present in 42% of *shams*^*Δ34*^ *Ser*^*rev6-1*^ double-mutant clones which cross the DV boundary (Fig 3D, 3D’ and 3G; n = 31). Since Delta is the only ligand remaining in these clones, these observations are in agreement with the conclusion that loss of Shams promotes Delta-mediated Notch activation. Next, we performed a similar analysis on *Delta* single-mutant and *Delta shams* double-mutant clones, where Serrate is the only remaining ligand. *Dl*^*RevF10*^ single-mutant clones crossing the boundary showed a ~65% penetrant loss of Cut expression (Fig 3E, 3E’ and 3G; n = 29). Similarly, *Dl*^*RevF10*^ *shams*^*Δ34*^ double-mutant clones which cross the DV boundary exhibited a ~64% penetrant loss of Cut expression (Fig 3F, 3F’ and 3G; n = 34). The comparable loss of Cut expression in *Dl*^*RevF10*^ and *Dl*^*RevF10*^ *shams*^*Δ34*^ clones suggests that loss of *shams* does not alter the activation of Notch by Serrate, which is the only remaining ligand inside these clones.

In addition to wing disc stainings, we examined the effects of *shams*^*Δ34*^, *Ser*^*rev6-1*^ and *Dl*^*RevF10*^ single-mutant and *shams*^*Δ34*^ *Ser*^*rev6-1*^ and *Dl*^*RevF10*^ *shams*^*Δ34*^ double-mutant clones on the adult wing size. Although the clones in these adult wings are not marked, inspection of 3rd instar wing imaginal discs indicates that *Ubx-FLP* induces sizeable clones in 100% of the discs. Adult wings harboring *shams*^*Δ34*^ clones do not exhibit any wing margin phenotypes, although some animals show wing vein loss (Fig 3I). Adult wings harboring clones of the *Ser*^*rev6-1*^ allele show severe wing scalloping (Fig 3J), in agreement with previous reports on this allele and other *Serrate* null alleles [36–38]. However, the degree of scalloping in adult wings harboring *shams*^*Δ34*^ *Ser*^*rev6-1*^ double-mutant clones is significantly less than that of the wings harboring *Ser* ^*rev6-1*^ clones (Fig 3K). Indeed, the average area of the wings harboring *Serrate* clones was significantly less than that of the wings harboring *Serrate shams* double-mutant clones (Fig 3N; *P*<0.05; n = 13 for each genotype). In contrast, the average area of wings harboring *Dl*^*RevF10*^ clones was not significantly different from the wings harboring *Dl*^*RevF10*^ *shams*^*Δ34*^ double mutant clones (Fig 3L–3N; *P*>0.05; n = 13 for each genotype). These results are in agreement with the data obtained from clonal analysis in larval wing discs and further support the notion that loss of *shams* partially suppresses the phenotypes caused by the loss of *Serrate*, likely by enhancing the Delta-mediated Notch activation in the developing wing.

### Loss of Shams augments *trans*-activation of Notch by ectopic Delta

**Fig 4:**
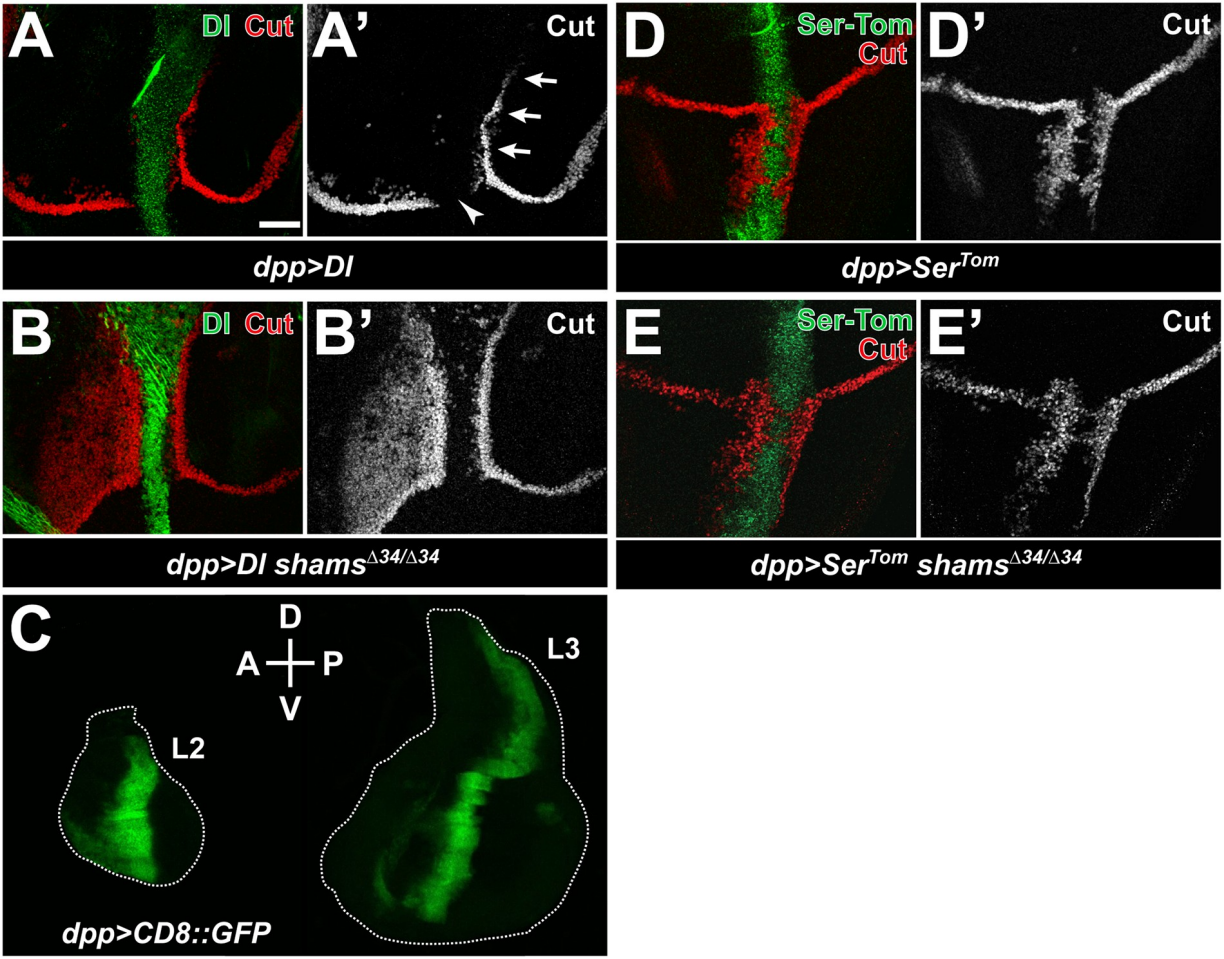
Loss of Shams augments *trans*-activation of Notch by ectopic Delta. (A-D’) Third instar wing imaginal discs raised at 25°C. Staining with anti-Delta antibody and Tomato fluorescence from the Serrate^Tom^ hybrid protein are shown in green; Cut staining is shown in red. Anterior is to the left, and dorsal is up. Scale bar in (A) is 25 μm and applies to all panels except C. (A-A’). Expression of Delta using *dpp-GAL4* (*dpp>Dl*) in a wild-type background. Arrows and the arrowhead indicate *trans*-activation and *cis*-inhibition, respectively. (B,B’) In a *shams*^*Δ34/Δ34*^ background, *dpp>Dl* induces broad activation of Cut anterior to the Dpp domain. (C) Wing imaginal discs from *dpp>CD8*::*GFP* second (L2) and third (L3) instar larvae are shown. Note the anterior broadening of the CD8::GFP signal in L2 compared to L3 disc. (D-D’) Expression of Serrate^Tom^ using *dpp-GAL4* (*dpp>Ser*^*Tom*^) in a wild-type background. (E-E’) Loss of *shams* does not alter Cut expression induced by *dpp>Ser*^*Tom*^.

In some cell types, the decision whether Notch signaling is activated or inhibited is determined based on the relative levels of Notch *trans*-activation and *cis*-inhibition by its ligands [6–8]. Given the complex interplay between *trans* and *cis* functions of ligands and potential feedback mechanisms, we used the GAL4-UAS system [39] to overexpress Delta or Serrate along the anterior-posterior boundary of the developing 3rd instar wing imaginal discs and assessed the effects of loss of *shams* on the *cis* and *trans* effects of each ligand on Notch. When raised at 25°C, animals overexpressing Delta within the Dpp expression domain (*dpp>Dl*) exhibit two phenotypes in wing imaginal discs: loss of the Cut-positive cells and disruption of the endogenous wing margin within the Dpp expression domain due to *cis*-inhibition (Fig 4A and 4A’; arrowhead), and ectopic Cut-positive cells that flank the Dpp expression domain in the dorsal-posterior quadrant due to *trans*-activation (Fig 4A and 4A’; arrows). In a *shams*^*Δ34/Δ34*^ mutant background, there is no obvious effect on *cis-*inhibition within the Delta-expressing stripe (Fig 4B and 4B’). However, we observe broad ectopic activation of Cut in a significant portion of the dorsal-anterior quadrant (Fig 4B and 4B’). These observations support the notion that loss of Shams promotes Delta-mediated *trans*-activation of Notch without affecting *cis*-inhibition. Of note, in late 2nd instar wing discs, the *dpp-GAL4* driver shows a much broader anterior expression domain compared to 3rd instar wing discs (Fig 4C), providing a likely explanation for the wide Cut expression domain observed in the dorsal-anterior quadrant of *dpp>Dl shams*^*Δ34/Δ34*^ 3rd instar discs.

To determine the effects of loss of Notch xylosylation on Serrate-mediated *trans*-activation and *cis*-inhibition of Notch, we performed similar overexpression experiments using a Tomato-tagged Serrate transgene (*UAS-Ser*^*Tom*^) [40]. As expected, overexpression of Ser^Tom^ results in *cis*-inhibition of Notch at the endogenous wing margin region and *trans*-activation of Notch outside of the Dpp expression domain in the ventral compartment (Fig 4D and 4D’). In contrast to *dpp>Dl*, no changes in Cut activation in or outside of the Dpp expression domain was observed between *dpp>Ser*^*Tom*^ and *dpp>Ser*^*Tom*^ *shams*^*Δ34/Δ34*^ animals (Fig 4D–4E’). These data indicate that in the developing wing disc, loss of Shams does not affect the *trans*-activation and *cis*-inhibition of Notch mediated by overexpressed Serrate.

### Notch *trans*-activation by *dpp>Dl* overcomes its *cis*-inhibition by endogenous ligands in *shams* mutants

**Fig 5:**
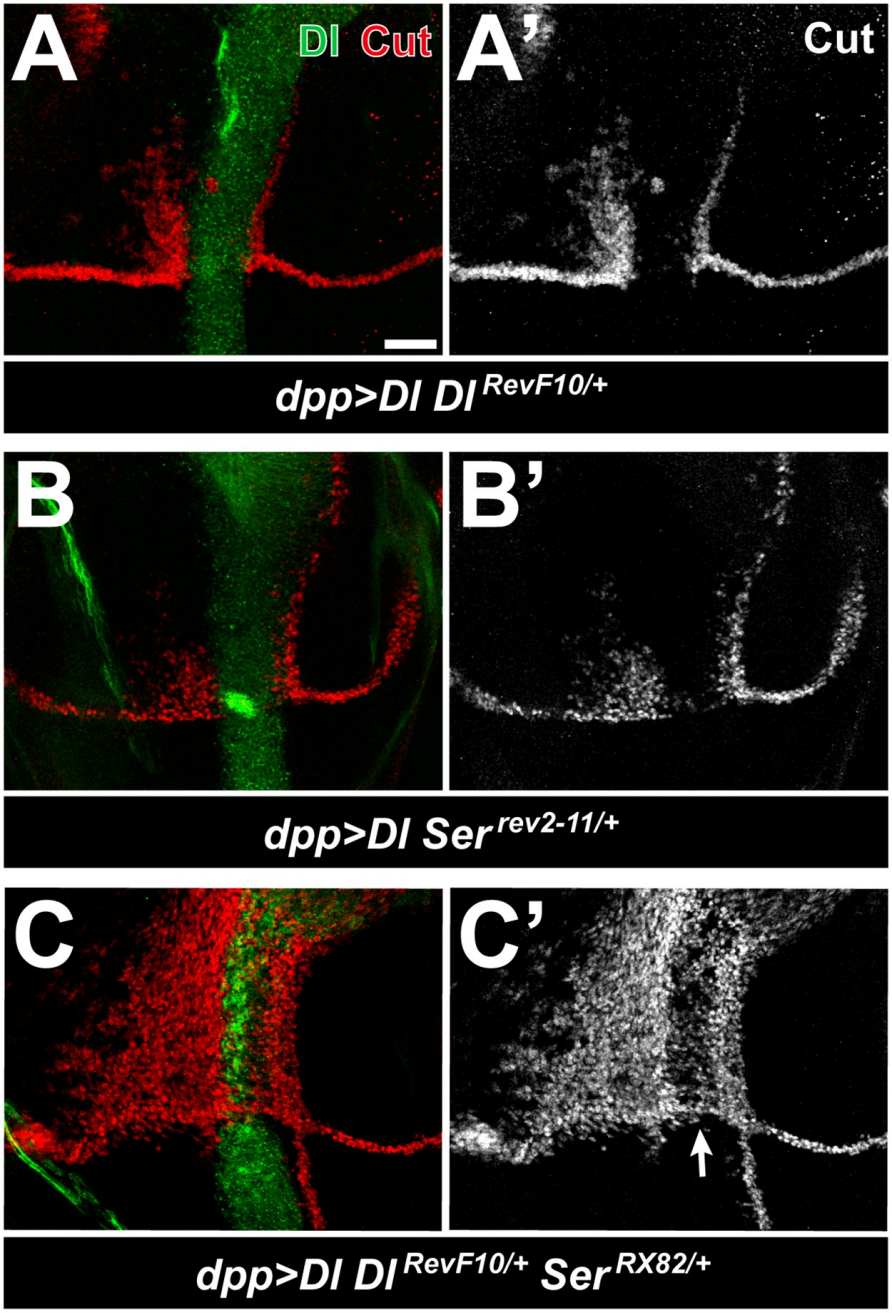
Reducing the level of endogenous ligands increases the *trans* activity of overexpressed Delta. (A-C’) Third instar larval wing discs raised at 25°C. Delta is visualized in green and Cut in red. Anterior is to the left, and dorsal is up. Scale bar = 25 μm. (A-B’) In a *Dl*^*RevF10/+*^ (A,A’) or *Ser*^*rev2-11/+*^ (B,B’) heterozygous background, *dpp>Dl* induces Cut activation anterior to the Dpp domain (compare to Fig 4A). (C,C’) *dpp>Dl Dl*^*+/–*^*Ser*^*+/–*^animals induce broad expression of Cut anterior to and within the Dpp domain and show some expansion of the ectopic Cut posterior to the Dpp domain. Cut expression in the wing margin is restored (arrow in C’).

Given the dramatic increase in the ability of *dpp>Dl* to *trans*-activate Notch upon loss of Shams, we decided to further explore the mechanism that prevents *dpp>Dl* from activating Notch in a *shams*^*+/+*^ background. In the sensory bristle lineage, decreasing the endogenous levels of Delta enhances the *trans*-activation of Notch by overexpressed Delta [9]. Accordingly, we examined the effects of removing one copy of endogenous *Delta* and/or *Serrate* on the expression of Cut in *dpp>Dl* larvae. In a *Dl*^*RevF10*^ or *Ser*^*rev2-11*^ heterozygous background, *dpp>Dl* induces moderate levels of Notch *trans*-activation in the dorsal-anterior quadrant (Fig 5A–5B’, compare to Fig 4A and 4A’). Moreover, in a *Dl*^*RevF10/+*^ *Ser*^*RX82/+*^ double-heterozygous background, *dpp>Dl* results in massive induction of Cut in the dorsal-anterior quadrant, broadening of the Cut expression domain in the dorsal-posterior quadrant, and appearance of Cut-expressing cells in the ventral-posterior compartment (Fig 5C and 5C’). In addition, Delta-mediated *cis-*inhibition is significantly decreased, because many cells in the Dpp expression domain express Cut and the endogenous wing margin is restored (Fig 5C and 5C’; arrow). These observations indicate that *cis*-inhibition of Notch by endogenous ligands potently limits the ability of overexpressed Delta to *trans*-activate Notch outside of the Dpp domain and cooperates with overexpressed Delta to *cis*-inhibit Notch inside of the expression domain, in agreement with the previous report by Jacobsen et al [9]. Therefore, loss of *shams* and decreasing endogenous ligands both result in a similar increase in the ability of *dpp>Dl* to *trans*-activate Notch in 3rd instar wing discs, suggesting that in *shams* mutants, Delta mediated *trans*-activation of Notch is able to overcome the *cis*-inhibitory effect of endogenous ligands.

### Cell-based aggregation and binding assays indicate increased binding between *trans*-Delta and Notch upon *shams* knockdown

**Fig 6:**
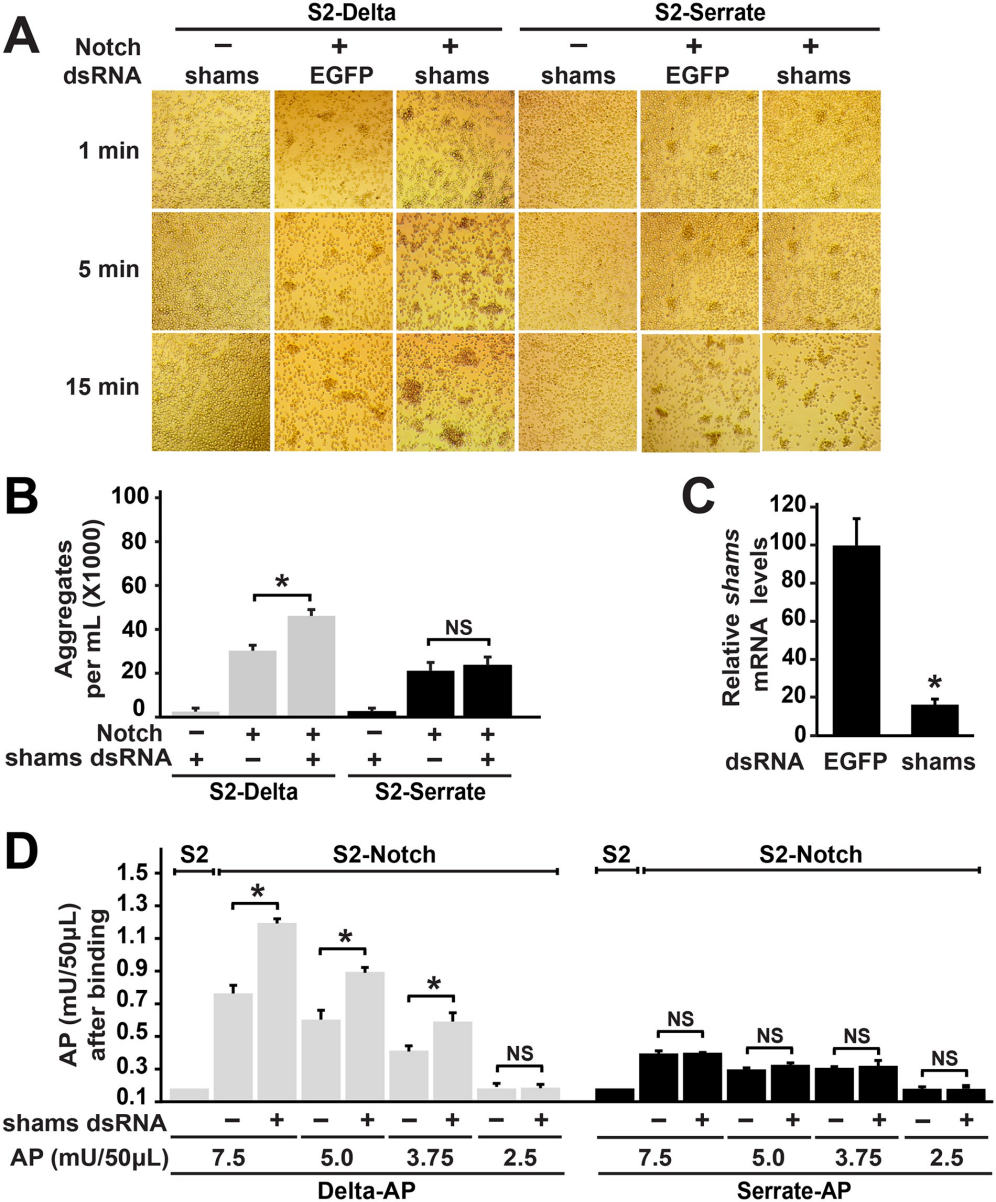
Cell-based aggregation and binding assays indicate increased binding between *trans*-Delta and Notch upon *shams* knockdown. (A) Representative images of co-culture assays between the indicated cell types at 1, 5 and 15 minutes are shown. (A) Shams KD in S2-N cells enhances their aggregation with S2-Dl cells but not with S2-Ser cells. (B) Quantification of number of cell aggregates greater than 6 cells after 5 minutes of co-culture. Error bars indicate standard error. Shams KD in S2-N cells increases the number of aggregates with S2-Dl cells but not with S2-Ser cells. *P<0.01, NS: not significant. (C) Relative *shams* mRNA levels are measured by qRT-PCR in S2 cells treated with either the control *EGFP* dsRNA or *shams* dsRNA. *P<0.01. (D) Graph shows the average AP (Delta-AP or Serrate-AP) units bound to S2 cells (control) and S2-N cells (treated with *EGFP* or *shams* dsRNA). Error bars indicate standard error. Shams KD in S2-N cells increases binding with Delta-AP but not with Serrate-AP. *P<0.01, NS: not significant.

To determine whether the effects of Shams on Delta-mediated signaling can be explained at the level of Notch-ligand binding, we performed S2 co-culture assays and assessed the effects of *shams* knock-down (KD) on the rate of aggregation formation and size of the aggregates formed between S2-Notch (S2-N) and S2-Delta (S2-Dl) or S2-Serrate^Tom^ (S2-Ser^Tom^) cells, which are indications for Notch-ligand binding [40,41]. For control experiments, we used plain S2 cells, which do not express Notch and Delta [41], and *EGFP* dsRNA. When co-cultured with plain S2 cells treated with *shams* dsRNA, S2-Dl cells form small aggregates which do not increase with time (Fig 6A). Co-culture of S2-Dl and control S2-N cells for one minute resulted in the formation of small aggregates, which increased in number and size as time elapsed (Fig 6A). Co-culture of S2-Dl with Shams KD S2-N cells formed much larger cell aggregates at 5 and 15 minutes (Fig 6A).Quantification of cell aggregates containing more than 6 cells at 5 minutes of co-culture showed that the number of aggregates was significantly higher when S2-N cells were incubated with *shams* dsRNA (Fig 6B; *P*<0.01). qRT-PCR experiments indicate that *shams* dsRNA decreases *shams* mRNA levels greater than 80% in S2 cells (Fig 6C). These observations suggest that Shams decreases the binding between Notch and Delta *in trans*. Of note, co-culture experiments between S2-Ser^Tom^ and S2-N cells showed that the size and the number of the S2-Ser^Tom^/S2-N aggregates did not change when *shams* levels were decreased (Fig 6A and 6B). These observations suggest that Shams KD does not affect the binding between Notch and *trans*-Serrate.

To more directly assess the effects of Shams KD on Notch-ligand binding, we used a quantitative receptor-ligand binding assay [23,42]. We incubated control and Shams KD S2-N cells with various concentrations of alkaline phosphatase (AP)-tagged ligand extracellular domains (Delta-AP or Serrate-AP) and asked whether Shams KD alters the binding of each ligand to S2-N cells in terms of the AP activity. Binding of each ligand-AP to plain S2 cells, which do not express Notch [41], was used as control. In agreement with the aggregation assays, treating S2-N cells with *shams* dsRNA significantly increased the binding of Delta-AP to these cells compared to *EGFP* dsRNA treated S2-N cells (Fig 6D). However, the amount of Serrate-AP bound to Shams KD and control S2-N cells was comparable at all four Serrate-AP concentrations tested (Fig 6D). These observations indicate that Shams negatively regulates Notch-Delta interaction but does not affect Notch-Serrate interaction.

Binding of Notch with *trans*-ligands can be affected by cell surface levels of Notch. Recent work has shown that combined loss of *O*-fucose and xylose residues from Notch alters its trafficking in 3rd instar wing discs, but loss of xylose by itself does not [43]. We have previously reported that loss of Shams or mutations that prevent the addition of xylose-glucose-*O* glycans to Notch EGF16-20 did not affect cell-surface expression of Notch in the 3rd instar wing discs, but enhanced the cell surface levels of Notch in the developing pupal wing disc [32]. Accordingly, we examined whether *shams* KD in S2 cells affects the cell surface expression of Notch. Detergent-free immunofluorescent staining of control and *shams* KD S2-N cells with an antibody against the Notch extracellular domain did not show any significant changes in the surface levels of Notch when *shams* is decreased (S2A and S2B Fig; n = 40 cells for each groups). Thus, the increased aggregation in Shams KD S2-N/S2-Dl co-cultures is not caused by an increase in Notch surface expression.

Although Serrate does not contain any predicted *O*-glucosylation sites, one of the Delta EGF repeats harbors the consensus *O*-glucosylation motif [44]. However, adding *shams* dsRNA to S2-Dl cells did not alter the size or the number of aggregates formed when cultured with S2-N cells (S3A and S3B Fig). We conclude that *shams* KD in Delta-expressing cells does not affect Notch-Delta *trans-*binding and that Shams functions in Notch-expressing cells. We also examined the effect of *shams* KD on surface distribution of Delta in S2-Dl cells using cell surface immunostaining with an antibody against the extracellular domain of Delta. *Shams* dsRNA treated S2-Dl cells did not show any significant change in surface level of Delta as compared to *EGFP* dsRNA treated S2-Dl cells (S3C and S3D Fig; n = 40 cells per group). This observation indicates that *shams* KD does not affect the surface level of Delta in S2 cells. We also examined whether loss of *shams* affects surface expression of Delta *in vivo*. Detergent-free staining of 3rd instar wing imaginal discs did not show any significant difference in surface expression of Delta between *shams*^*Δ34*^ mutant clones and the neighboring wild-type and heterozygous cells (S3E Fig; n = 12 clones), indicating that Shams does not regulate the surface levels of Delta in 3rd instar wing imaginal discs.

### Shams knockdown does not alter the inhibitory effect of *cis*-ligands on cell aggregation

**Fig 7:**
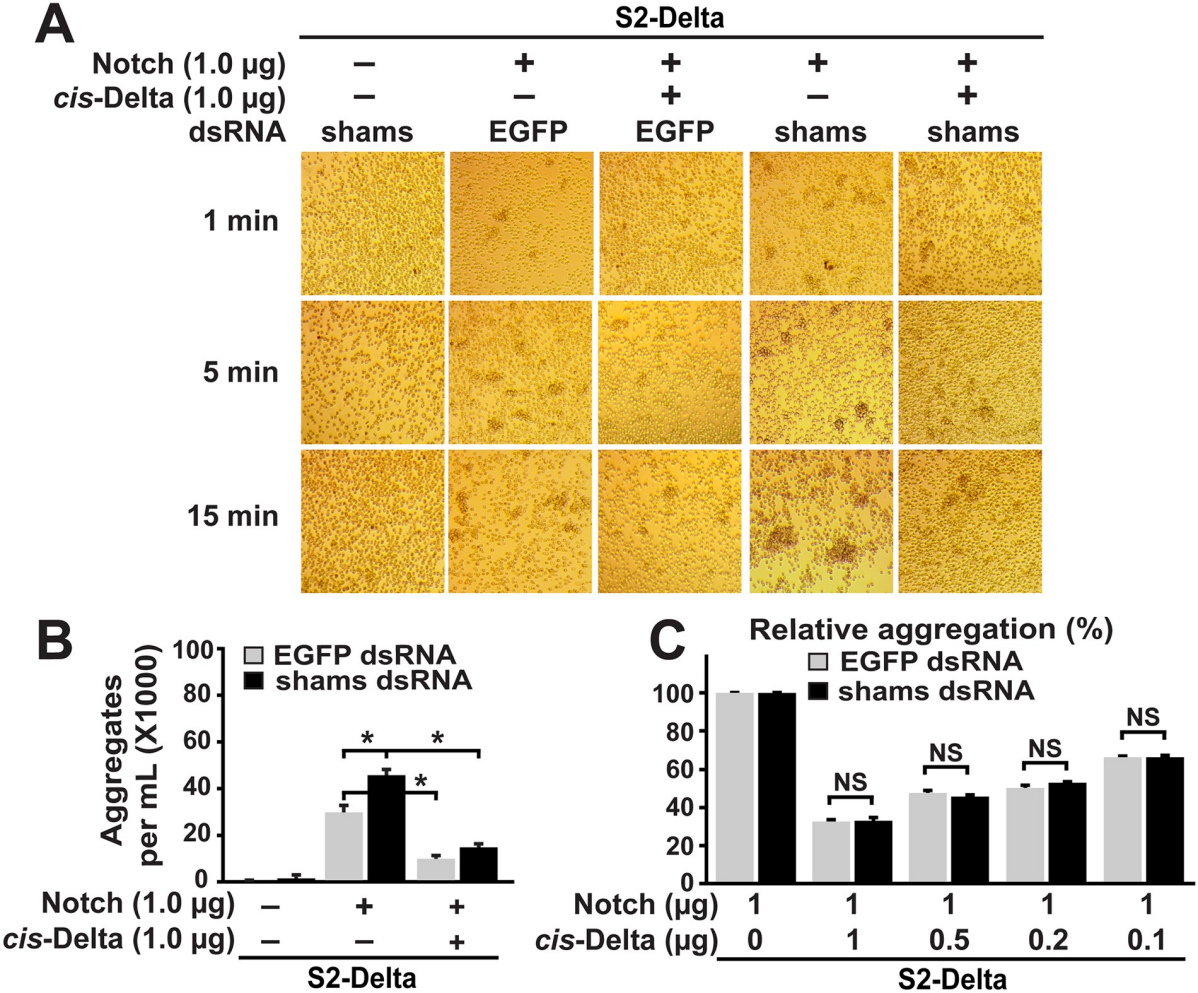
Shams knockdown does not alter the inhibitory effect of *cis*-Delta on cell aggregation mediated by Notch and *trans*-Delta. (A) Co-expression of Delta and Notch in a 1:1 ratio in S2 cells significantly decreases their ability to aggregate with S2-Dl cells. Shams KD does not affect the ability of Notch to respond to *cis*-Delta. (B) Quantification of number of cell aggregates greater than 6 cells after 5 minutes of co-culture. Error bars indicate standard error. *P<0.01. (C) Relative aggregation between S2-Dl cells and S2 cells co-transfected with indicated ratios of Notch and *cis*-Delta expression constructs. Number of cell aggregates greater than 6 cells after 5 minutes of co-culture were quantified for each combination and shown as a percentage of the number of aggregates between S2-Delta and S2-N^transient^ cells in the absence of *cis*-Delta. Error bars indicate standard error. NS: not significant.

**Fig 8:**
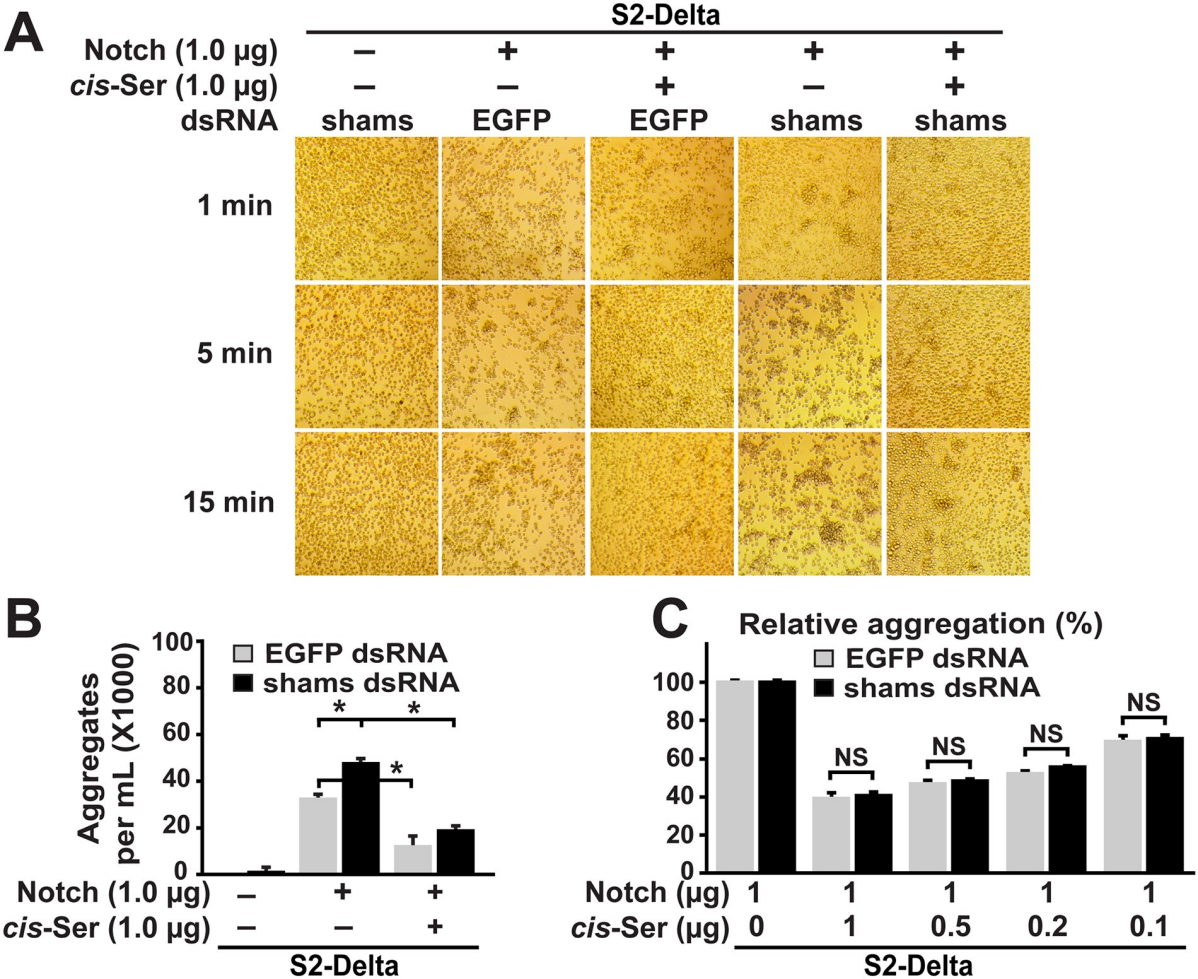
Shams knockdown does not alter the inhibitory effect of *cis*-Serrate on cell aggregation mediated by Notch and *trans*-Delta. (A) Co-expression of Serrate and Notch in a 1:1 ratio in S2 cells significantly decreases their ability to aggregate with S2-Dl cells. Shams KD does not affect the ability of Notch to respond to *cis*-Serrate. (B) Quantification of number of cell aggregates greater than 6 cells after 5 minutes of co-culture. Error bars indicate standard error. *P<0.01. (C) Relative aggregation between S2-Dl cells and S2 cells co-transfected with indicated ratio of Notch and *cis*-Serrate expression constructs. Number of cell aggregates greater than 6 cells after 5 minutes of co-culture were quantified for each combination and shown as a percentage of the number of aggregates between S2-Delta and S2-N^transient^ cells in the absence of *cis*-Serrate. Error bars indicate standard error. NS: not significant.

To determine whether Shams modulates binding between Notch and *cis*-ligands, we asked whether *shams* KD affects the ability of ligands co-expressed with Notch to decrease the aggregation between the Notch-expressing cells and S2-Dl cells. To this end, we performed aggregation assays between S2-Dl cells and S2 cells transiently transfected with a Notch expression vector as control (S2-N^transient^) or equal amounts of Notch and *cis*-ligand expression vectors (S2-N&Dl^transient^ or S2-N&Ser^transient^). The relative aggregation between S2-Dl and S2-N^transient^ cells in the presence and absence of *cis*-ligands was used as an indication for the degree of *cis*-inhibitory effect of each ligand. For example, if the number of aggregates between S2-Dl and S2-N&Ser^transient^ cells is 25% of the number of aggregates between S2-Dl and S2-N^transient^ cells (i.e. relative aggregation 25%), we would conclude that *cis-*Serrate was able to block the interaction between Notch and *trans*-Delta by 75%. Co-culture of S2-Dl and control S2-N^transient^ cells formed small aggregates, which grew in size and number with time, similar to the co-culture between S2-Dl and stable S2-N cells (Figs 7A and 8A, compare to Fig 6A). The aggregation was dramatically decreased when S2-Dl cells were co-cultured with S2-N&Dl^transient^ or S2-N&Ser^transient^ cells (Figs 7A, 7B, 8A and 8B). This is most likely due to *cis*-inhibition of Notch by ligands expressed in the same cell, as shown previously in *Drosophila* and mammalian cell-culture assays [11,40]. Co-culture of S2-Dl cells and *shams* KD S2-N^transient^ cells formed aggregates more quickly and resulted in larger aggregates, recapitulating the results seen with stable S2-N cells (Figs 7A and 8A). Addition of *cis*-ligands to these cells (*shams* KD S2-N&Dl^transient^ or S2-N&Ser^transient^) also decreased their aggregation with S2-Dl cells (Figs 7A, 7B, 8A and 8B). Importantly, quantification of aggregates after five minutes of co-culture showed that the magnitude of this *cis-*inhibition was comparable to the *cis*-inhibition observed in co-culture between S2-Dl and control S2-N&Dl^transient^ or S2-N&Ser^transient^ cells, which were incubated with *EGFP* dsRNA instead of *shams* dsRNA (Figs 7C and 8C). These observations suggest that *shams* KD does not diminish the ability of *cis*-ligands to oppose the binding between Notch and *trans*-Delta.

One caveat of this experiment is that the relative level of *cis-*ligands expressed in these cells might be too high and therefore be able to inhibit *trans*-Delta/Notch binding irrespective of *shams* levels. To address this concern, we performed three additional sets of aggregation assays for each *cis*-ligand and successively decreased the ratio of *cis*-ligand expression plasmid to the Notch expression plasmid used in each set (0.5:1, 0.2:1, 0.1:1). To ensure similar baseline levels of *trans*-Dl/Notch binding, we used the same amount of Notch expression plasmid in all experiments and only changed the amount of *cis*-ligand expression plasmids. Aggregation between S2-Dl and S2-N^transient^ cells was once again inhibited by expression of *cis*-ligands (both *cis-*Delta and *cis-*Serrate) even at lowest levels. Quantitation of the number of cell aggregates greater than six cells at five minutes of co-culture showed that the magnitude of inhibition by *cis*-ligands was concentration-dependent: the lower the ratio of *cis*-ligand to Notch expression plasmid, the lower the ability of *cis*-ligands to decrease the number of aggregates between S2-Dl and S2-N^transient^ cells (Figs 7C and 8C). These data indicate that the level of *cis*-ligands used in our assays are not saturating. Under each *cis*-ligand/Notch ratio, the *cis*-inhibitory effect of each ligand on S2-Dl/S2-N^transient^ aggregation was almost identical in cells treated with *EGFP* dsRNA and *shams* dsRNA (Figs 7C and 8C). These observations indicate that *shams* KD does not decrease the ability of *cis*-ligands to block the interaction between *trans*-Delta and Notch, and suggest that Shams does not affect the binding between Notch and *cis-*ligands. Taken together, these cell culture data support the conclusion that loss of Shams specifically enhances the binding of Notch with *trans*-Delta without affecting its binding with *cis-*ligands.

## Discussion

Modifications of the extracellular domain of Notch receptor by glycosylation influence its activity in different contexts [20–22]. Xylosylation of the Notch receptor by the glycosyltransferase enzyme Shams has been reported to negatively regulate Notch signaling in *Drosophila* [32]. Loss of Notch xylosylation is associated with increased Notch expression at the cell surface in pupal wing disc but not in the larval wing imaginal discs [32]. However, the mechanisms underlying the tissue-specific phenotypes of *shams* and the effects of xylosylation on other steps of Notch signaling such as ligand binding and *cis*-inhibition are not known. In the present study, we provide several lines of evidence suggesting that the wing vein loss phenotype observed upon loss of Notch xylosylation results from a specific increase in Delta-mediated Notch activation. First, adding one copy of *Delta* enhances wing vein loss phenotype in *shams* mutant animals. Second, a *Notch* transgene with mutations in functional xylosylation sites results in wing vein loss when combined with an extra copy of *Delta*. Third, removing one copy of *Delta* suppresses the wing vein phenotype in *shams* mutant animals. Last, Shams KD in S2-N cells enhances their binding with AP-tagged Delta and their aggregation with S2-Dl cells without affecting cell surface levels of Notch. Since functional xylose residues reside in EGF16-20 of the Notch receptor [32], our work identifies the glycosylation of this domain as a novel mechanism for the modulation of Delta-mediated Notch activation in *Drosophila*. Although Notch EGF11-12 are required for binding of Notch to both ligands [2], a mutation in Notch EGF8 affects Notch-ligand binding and signaling in a ligand-selective manner [42]. Together with this report [42], our data suggest that distinct EGF repeats other than EGF11-12 are involved in preferential or exclusive modulation of the response of Notch to its ligands. Moreover, the differential effects of decreasing Notch xylosylation on the binding and response of Notch to *trans*-Delta versus *cis*-ligands suggest different domains or structural conformations of Notch might be involved in binding to *trans-* versus *cis*-ligands.

The increased Delta-mediated signaling upon loss of *shams* can theoretically be due to increased *trans*-activation, decreased *cis*-inhibition, or both. If the primary mechanism for activation of Notch signaling and loss of wing vein in *shams* mutants were decreased *cis*-inhibition by Delta, removing one copy of *Delta* should have enhanced the wing vein loss phenotype in *shams* mutants. On the contrary, the *shams* wing vein loss was suppressed upon *Delta* heterozygosity. Moreover, loss of *shams* dramatically enhances Notch *trans*-activation but does not affect Notch *cis*-inhibition by Delta in *dpp>Dl* animals, and *shams* KD promotes S2 cell aggregation mediated by Notch and *trans*-Delta without decreasing the inhibitory effect of *cis*-Delta on Notch in these assays. Therefore, although it is still possible that Shams plays a minor role in the interaction of Notch with *cis*-ligands in certain contexts, our data strongly suggest that the primary mechanism for the wing vein loss in *shams* mutants is an increase in Notch *trans*-activation by Delta, not a decrease in Notch *cis*-inhibition by Delta.

In line with a previous report in the bristle lineage [9], we find that removing one copy of *Delta* and/or *Serrate* in *dpp>Dl* animals results in ectopic activation of Notch in the dorsal-anterior quadrant, indicating that *cis*-inhibition by endogenous ligands normally opposes the *trans*-activation of Notch by ectopic Delta in this region. These observations are in agreement with quantitative analyses indicating that the balance between the activity of *trans*- and *cis*-ligands determines whether a given cell assumes a signal-receiving state or not [11,12]. A similar increase in Delta-mediated *trans*-activation of Notch is seen in *dpp>Dl* animals upon loss of Shams despite the presence of endogenous ligands. Accordingly, we propose that Shams functions to regulate the balance between *trans*-activation of Notch by Delta and *cis*-inhibition of Notch by ligands, and that in the absence of shams, *trans*-activation of Notch by Delta overcomes the *cis*-inhibitory effects of ligands. Our cell aggregation assays suggest that Shams mediates this role by impeding the ability of Notch to bind Delta in *trans*, without altering the binding of Notch to *cis*-ligands. As shown in Fig 1, increasing the gene dosage of *Delta* by itself or combined with an additional copy of wild-type *Notch* does not result in wing vein loss, likely because the balance between the Notch and Delta levels and also the balance between the *trans* and *cis* activities of Delta are preserved. Based on our model, loss of xylose residues on Notch due to loss of Shams or mutations in biologically-relevant Shams target sites on Notch tips the balance between *trans-* and *cis-* activities of Delta in favor of *trans*-Delta, as evidenced by the net gain of Notch signaling and loss of wing vein in animals with three copies of *Delta* and simultaneous loss of Notch xylosylation.

Both Shams and Fringe regulate Notch signaling by adding carbohydrate residues to *O*-linked monosaccharides on Notch EGF repeats and generating disaccharides: xylose-glucose-*O* in the case of Shams, and GlcNAc-fucose-*O* in the case of Fringe [23,25,32,45]. Moreover, as shown here for Shams and previously for Fringe [23,46], both proteins regulate Notch-ligand interactions. However, the effects of these enzymes on the binding and response of Notch to Delta versus Serrate and to *trans*-Delta versus *cis*-Delta seem to be distinct from each other. Fringe promotes Delta-mediated *trans*-activation and simultaneously decreases Serrate-mediated *trans*-activation of Notch [26]. Moreover, it has recently been shown that Fringe proteins affect the *trans* and *cis* interaction of Notch with each ligand in the same direction, i.e., they promote Notch-Delta interactions both in *cis* and in *trans*, and inhibit Notch-Serrate interaction both in *cis* and in *trans* [11]. In contrast, our data indicate that Shams decreases the binding of Notch to and its activation by *trans*-Delta without affecting its interactions with *cis-*ligands. Further, our aggregation assays and most of our *in vivo* observations indicate that binding of Notch to and its activation by *trans*-Serrate is not significantly affected by Shams, although the appearance of a low-penetrance wing margin loss phenotype in *shams*^*–/–*^*Serrate*^*+/–*^animals suggests that Shams might play a redundant role in Serrate-induced Notch signaling in some contexts. The functional differences between Shams and Fringe likely explain their distinct mutant phenotypes in the wing, i.e. loss of wing vein in the case of *shams* and wing vein thickening and loss of wing margin in the case of *fringe* mutants [47]. The distinct roles of Fringe and Shams in regulating Notch signaling along with the differentially distributed EGF repeats with Shams (xylose) versus Fringe (GlcNAc) elongation across the Notch extracellular domain [32,48] suggest that the combined function of the sugar modifications mediated by these enzymes ensures optimal level of Notch pathway activity in several contexts during fly development.

## Materials and methods

### *Drosophila* strains and genetics

The following strains were used in this study: *y w*, *y w; D/TM6*, *Tb*^*1*^, *w; noc*^*Sco*^*/CyO*, *w; noc*^*Sco*^*/CyO; TM3*, *Sb*^*1*^*/TM6*, *Tb*^*1*^, *dpp-GAL4*, *Df(3R)BSC494/TM6C*, *Sb*^*1*^, *Dl*^*9P*^*/TM6*, *Tb*^*1*^, *Dl*^*RevF10*^ *Ser*^*RX82*^*/TM6*, *Tb*^*1*^, *UAS-CD8*::*GFP* (Bloomington *Drosophila* Stock Center), *shams*^*Δ34*^*/TM6*, *Tb*^*1*^ [32], *PBac{N*^*gt-wt*^*}VK22*, *PBac{N*^*gt-16_20*^*}VK22*, [28], *P{Dl*^*gt-wt*^*}attP2*, *PBac{Dl*^*gt-wt*^*}VK37*, *PBac{Ser*^*gt-wt*^*}VK37* [11], *y w Ubx-FLP tub-GAL4 UAS-GFP*^*nls*^*-6X-Myc; FRT82B y*^*+*^ *tub-GAL80/TM6*, *Ubx* [27], *w; UAS-Dl* (Gary Struhl), *Dl*^*RevF10*^*/TM6*, *Tb*^*1*^ [49], *Ser*^*rev6-1*^*/TM6*, *Tb*^*1*^ [50], *Ser*^*rev2-11*^*/TM6*, *Tb*^*1*^ [50], *Ser*^*RX106*^ [51], *UAS-Ser*^*wt*^*-Tomato* (*UAS-Ser*^*Tom*^) [40], *FRT82B shams*^*Δ34*^ *Ser*^*rev6-1*^*/TM6*, *Tb*^*1*^, *Dl*^*9P*^ *shams*^*Δ34*^*/TM6*, *Tb*^*1*^, *FRT82B shams*^*Δ34*^ *Dl*^*RevF10*^*/TM6*, *Tb*^*1*^, *FRT82B Dl*^*RevF10*^*/TM6*, *Tb*^*1*^, *dpp-GAL4 shams*^*Δ34*^*/TM6*, *Tb*^*1*^, *UAS-Dl; shams*^*Δ34*^*/TM6b*,*Tb*^*1*^, *UAS-Ser*^*Tom*^*/CyO; shams*^*Δ34*^*/TM6b*, *Tb*^*1*^, *y w Ubx-FLP/FM7; FRT82B Sb*^*63*^ *y*^*+*^*/TM6*, *Ubx* (this study).

### *Drosophila* genetics

All crosses were performed on standard media. All crosses were incubated at 25°C except for adult wings listed at 30°C, which were incubated at 25°C until late larval stage and shifted to 30°C during pupal stages. To generate MARCM clones [52] in 3rd instar wing discs, *y w Ubx-FLP tub-GAL4 UAS-GFP*^*nls*^*-6X-Myc; FRT82B y*^*+*^ *tub-GAL80/TM6*, *Ubx* females were crossed to *FRT82B mutant/TM6*, *Tb*^*1*^ males, wherein mutant stands for *shams*^*Δ34*^, *Ser*^*rev6-1*^, *Delta*^*RevF10*^, *Delta*^*RevF10*^ *shams*^*Δ34*^, or *shams*^*Δ34*^ *Ser*^*rev6-1*^. Crosses were kept at 25°C and mosaic *Tb*^*+*^ 3rd instar larvae were selected for dissection. To generate clones in adult wings, *FRT82B shams*^*Δ34*^*/TM6*, *Tb*^*1*^, *FRT82B Ser*^*rev6-1*^*/TM6*, *Tb*^*1*^, *FRT82B shams*^*Δ34*^ *Ser*^*rev6-1*^*/TM6*, *Tb*^*1*^, *FRT82B Delta*^*RevF10*^*/TM6*, *Tb*^*1*^, and *FRT82B Delta*^*RevF10*^ *shams*^*Δ34*^*/TM6*, *Tb*^*1*^ males were crossed to *y w Ubx-FLP/FM7; FRT82B Sb*^*63*^ *y*^*+*^*/TM6*, *Ubx* females and raised at 25°C. *Sb*^*63*^, *Tb*^*+*^ flies were selected for scoring the wings. All of the scored flies had regions of *Sb*^*+*^ microchaete on the thorax, confirming the generation of mutant clones in wing imaginal discs. *dpp>Dl shams*^*Δ34/Δ34*^ animals were generated by crossing animals harboring a *dpp-GAL4 shams*^*Δ34*^ recombinant chromosome to *UAS-Dl; shams*^*Δ34*^*/TM6b*,*Tb*^*1*^ animals. *Tb*^*+*^ 3rd instar larvae were selected for dissection. To generate *dpp>Ser*^*Tom*^ *shams*^*Δ34/Δ34*^ animals, *dpp-GAL4 shams*^*Δ34*^ animals were crossed to *UAS-Ser*^*Tom*^*/CyO; shams*^*Δ34*^*/TM6b*, *Tb*^*1*^ animals. *Tb*^*+*^ 3rd instar larvae expressing Tomato were selected for dissection. To examine the expression pattern of cDNAs driven by *dpp-GAL4* in larval wing imaginal disc, *dpp-GAL4/TM6*, *Tb*^*1*^ males were crossed to *UAS-CD8*::*GFP* females. Late 2nd and late 3rd instar *Tb*^*+*^ larvae were selected and dissected.

### Dissections, staining, image acquisition and processing

Dissection and staining were performed by using standard methods. For surface staining, S2 cells were incubated with antibodies against the Notch extracellular domain (NECD) or the Delta extracellular domain (Dl-ECD) in the absence of detergent. A similar detergent-free protocol was used for Delta surface staining of wing imaginal discs. Antibodies used were mouse α-Cut (2B10) 1:500, mouse anti-Wg (4D4) 1:100, mouse anti-NECD (C458.2H) 1:100 (all from DSHB), guinea pig anti-Dl-ECD 1:3000 (Gift from M. Muskavitch) [15], goat α-mouse-Cy3 1:500, donkey α-mouse-Cy5 1:500 and donkey α-Guinea Pig-Cy3 1:500 (Jackson ImmunoResearch Laboratories). Adult wings were imaged using Zeiss Axioscope-A1 and Nikon Ci-L upright microscopes. Wing areas were measured in term of square pixels using ImageJ 1.47. Confocal images were scanned using a Leica TCS-SP5 microscope and processed with Amira5.2.2. Images were processed with Adobe Photoshop CS5; Figures were assembled in Adobe Illustrator CS5.

### S2 culture, ligand binding and cell aggregation assays

S2 cells (Invitrogen) were cultured in Schneider's *Drosophila* Medium (Lonza) supplemented with 10% fetal bovine serum and penicillin-streptomycin (100 U/mL). For S2-N and S2-Dl stable cell lines (DGRC, Bloomington, USA), 200 nM methotrexate (Sigma-Aldrich) was added. S2-Ser^Tom^ stable cells [40] were cultured in M3 medium (Gibco) supplemented with 10% fetal bovine serum and 100 μg/mL of hygromycin B. For knock-down, 7.5 μg of either *EGFP* dsRNA (control) or *shams* dsRNA was added to the culture medium of S2, S2-N or S2-Dl cells, and the cells were cultured for 24 hours at 25°C prior to induction with CuS0_4_ (0.7 mM).

Ligand-receptor binding assays were performed as described previously [42] with minor modifications. In brief, S2 cells (2 x 10^6^ cells/well) were transiently transfected with the constructs expressing the extracellular domain of Delta or Serrate fused to alkaline phosphatase (6.0 μg of either *pMT-Delta*-AP or *pMT-Serrate-*AP per well). After 24 hours, the transfected cells were induced by adding 0.7mM CuSO_4_ to the media. After three days of induction, conditioned media were collected and the amount of AP was determined by quantifying AP activity in each medium by following the manufacturer’s instructions (Phospha-Light System, Applied Biosystems) and using FLUOstar OPTIMA (BMG Labtech). In parallel, plain S2 or S2-N cells (2 x 10^6^ cells/well) were induced by 0.7mM CuSO_4_ after 24 hours of treatment with *EGFP* dsRNA or *shams* dsRNA. After three days of induction, cells (S2 or S2-N cells) were collected and incubated with conditioned media (containing defined concentrations of Delta-AP or Serrate-AP) for 90 minutes on rotary shaker. After three times washing and cell lysis, the endogenous AP activity was heat inactivated (60°C for 10 min). The amount of AP-tagged ligand bound to S2-N cells was assayed as per the manufacturer’s instructions (Phospha-Light System, Applied Biosystems) and using FLUOstar OPTIMA (BMG Labtech). Binding of each ligand (Delta-AP or Serrate-AP) with plain S2 cells (which lack endogenous Notch protein [41]) was used as control. Experiments were performed in triplicate and repeated three times.

Cells were incubated with CuSO_4_ for 1–2 days and then used in aggregation assays. For each aggregation assay, 2.5 x 10^5^ of the dsRNA-treated cells (S2, S2-N, S2-Dl) were mixed with 5 x 10^5^ S2-Dl, S2-Ser^Tom^ or S2-N cells (induced with 0.7 mM CuSO_4_ for 3 hours prior to co-culture) in a total volume of 200 μl medium in a 24-well plate.

For co-culture assays expressing *cis-*ligands, after 24 hours of treatment with either *EGFP* or *shams* dsRNA, S2 cells were transiently transfected with 2 μg total of either *pBluescript* (control), *pBluescript* and *pMT-Notch* (S2-N^transient^), *pMT-Notch* and *pMT-Delta* (S2-N&Dl^transient^), or *pMT-Notch* and *pMT-Serrate* (S2-N&Ser^transient^) using 3 μL of FuGENE HD (Promega). One μg of *pMT-Notch* was used in all assays. The remaining 1 μg was either *pBluescript* alone, *pMT-Delta* alone, *pMT-Serrate* alone, or a mixture of 0.5 μg, 0.2 μg or 0.1 μg of *pMT-Delta* or *pMT-Serrate* and *pBluescript* (Figs 7C and 8C). For aggregation assays, 2.5 x 10^5^ dsRNA treated cells were mixed with 5 x 10^5^ S2-Dl cells (induced for 3 hours prior to co-culture with CuSO_4_) in a total volume of 200μL in a 24-well plate. Co-cultured cells were gently shaken at 150 rpm to allow aggregation. Images of aggregate formation were taken at reported time points. The number of cell aggregates was quantified using a hemocytometer after 5 minutes of co-culture. Each assay was repeated at least three times. *P*-values were determined either by Student’s *t*-test or by One-Way ANOVA with Tukey’s multiple comparisons test.

### Generation of dsRNA for S2 aggregation assays

*EGFP* dsRNA primers:
Forward primer-GAAATTAATACGACTCACTATAGGGGGTGAGCAAGGGCGAGGAG
Reverse primer-GAAATTAATACGACTCACTATAGGGGGTCTTTGCTCAGGGCGG

*shams* dsRNA Primers:
Forward primer-TTAATACGACTCACTATAGGGGAGATGCTGTATGTGGACACGGAT
Reverse Primer-TTAATACGACTCACTATAGGGGAGATCCGTGGATAACCTTAACGA

Template DNAs were generated by PCR amplification from *pAct-EGFP* plasmid DNA or *y w* genomic DNA. Purified PCR products were used as template for *in vitro* transcription reactions using the T7 MEGAscript kit (Ambion). Double-stranded RNA was purified with RNeasy Mini kit (Qiagen).

### qRT-PCR assays

*shams* and *Rp49 (RpL32)* mRNA expression in S2 cells treated with either *EGFP* or *shams* dsRNA were assayed by qRT-PCR using TaqMan One-Step RT-PCR Master Mix and TaqMan primers/probe sets (Life Technologies Dm02144576_g1 and Dm02151827_g). Relative *shams* mRNA levels were compared using the 2^*–*ΔΔCT^ method. *P*-values were determined by Student’s *t*-test.

## Supporting information

S1 Fig Removing one copy of *Serrate* results in a low penetrance wing margin loss phenotype upon loss of shams. (A-B) All animals raised at 25°C. (A) Wing margin loss is observed in 21% of *Ser*^*rev6-1/+*^ *shams*^*Δ34/Δ34*^ animals (n = 73). (B) Adding one copy of a *Serrate* genomic transgene rescues the wing margin loss in these animals (n = 31).
(TIF)

S2 Fig Knock-down of Shams in S2-N cells does not affect the cell surface expression of Notch. (A) Representative images showing cell surface Notch expression (red) in S2 cell (control) and S2-N cells (treated with *EGFP* or *shams* dsRNA). No difference in expression levels is apparent. Scale bar = 25 μm. (B) Graph shows the average fluorescence intensity in dsRNA (*EGFP* or *shams*) treated S2-N cells (n = 40 cells in each group). Error bars indicate standard error. NS: not significant.
(TIF)

S3 Fig Knock-down of Shams in S2-Dl cells does not alter their aggregation with S2-N cells and the cell surface expression of Delta. (A) Cell aggregation assays were performed between S2-N cells with S2 cells treated with *shams* dsRNA (Shams KD), S2-Dl cells treated with a control dsRNA (*EGFP*) or S2-Dl cells treated with *shams* dsRNA. Representative images of each co-culture at 1, 5 and 15 minutes are shown. (B) Quantification of number of cell aggregates greater than 6 cells after 5 minutes of co-culture. Error bars indicate standard error. Note that Shams KD does not change the number of aggregates significantly (*P>*0.05). NS: not significant. (C) Representative images showing cell surface Delta expression (red) in S2 cells (control) and S2-Dl cells (treated with *EGFP* or *shams* dsRNA). No difference in expression levels is apparent. Scale bar = 25 μm. (D) Graph shows the average fluorescence intensity of surface Delta in dsRNA (*EGFP* or *shams*) treated S2-Dl cells (n = 40 cells for each group). Error bars indicate standard error. NS: not significant. (E) Detergent-free immunostaining for Delta extracellular domain (Dl-ECD) in third instar wing imaginal discs harboring *shams*^*Δ34*^ MARCM clones (marked with GFP; n = 12). No difference in surface level of Delta between wild-type and mutant cells is apparent. Scale bar = 25 μm.
(TIF)
