# Supplementary figures and images for: Xylosylation of the Notch receptor preserves the balance between its activation by trans-Delta and inhibition by cis-ligands in Drosophila
Source: PLoS Genet. 2017 Apr 10;13(4):e1006723. doi: 10.1371/journal.pgen.1006723 (PMC5402982; doi:10.1371/journal.pgen.1006723)

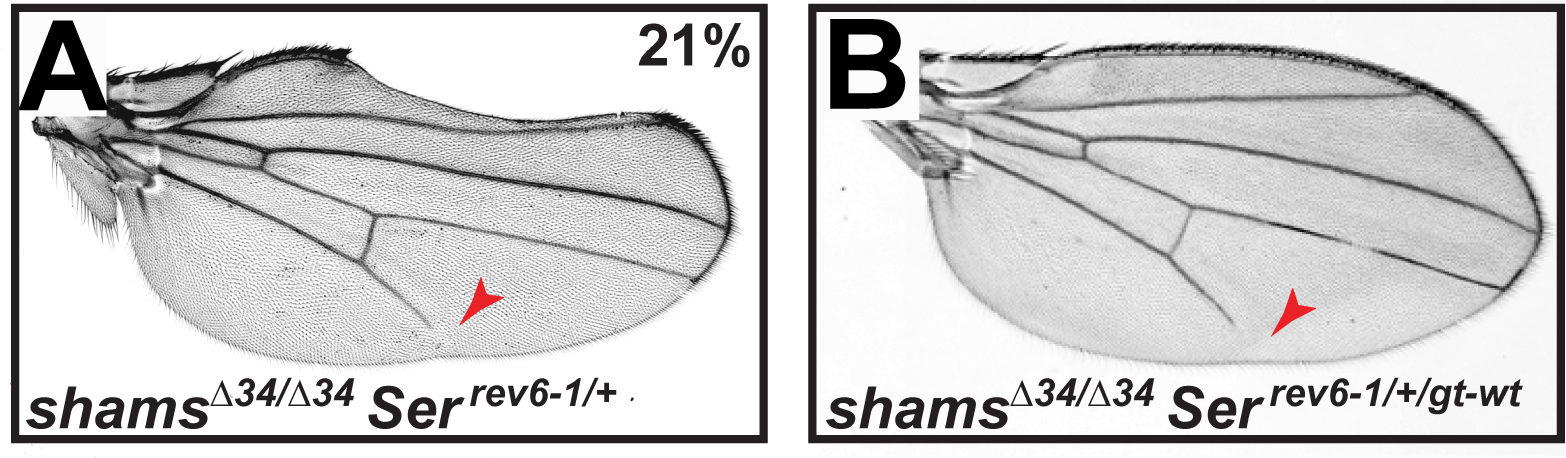

Supplement: S1 Fig — (A-B) All animals raised at 25°C. (A) Wing margin loss is observed in 21% of Serrev6-1/+ shamsΔ34/Δ34 animals (n = 73). (B) Adding one copy of a Serrate genomic transgene rescues the wing margin loss in these animals (n = 31). (TIF) [file pgen.1006723.s001.tif]

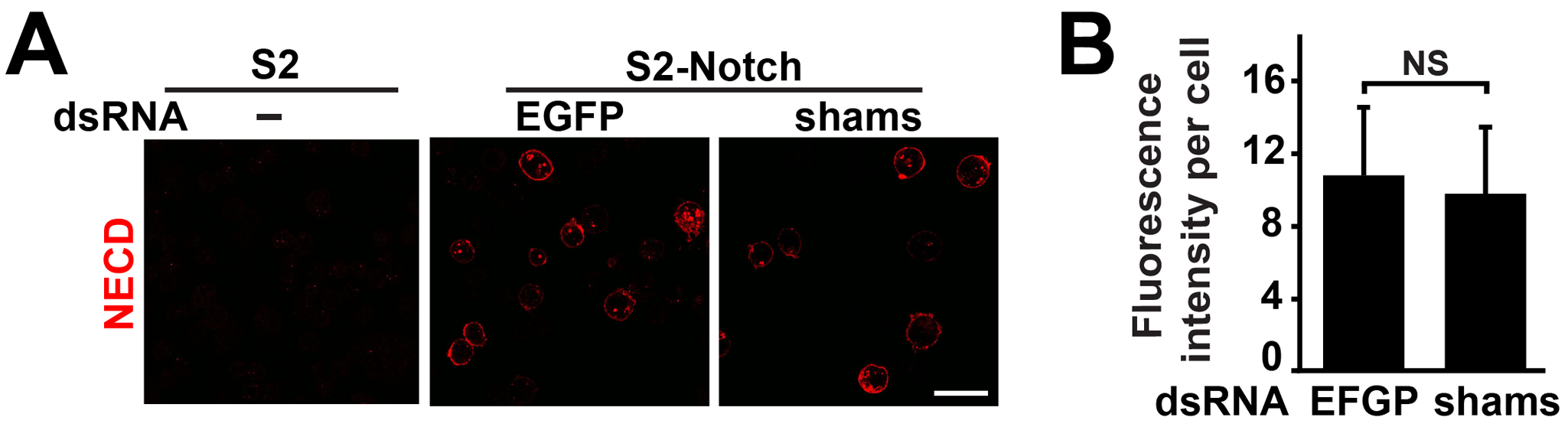

Supplement: S2 Fig — (A) Representative images showing cell surface Notch expression (red) in S2 cell (control) and S2-N cells (treated with EGFP or shams dsRNA). No difference in expression levels is apparent. Scale bar = 25 μm. (B) Graph shows the average fluorescence intensity in dsRNA (EGFP or shams) treated S2-N cells (n = 40 cells in each group). Error bars indicate standard error. NS: not significant. (TIF) [file pgen.1006723.s002.tif]

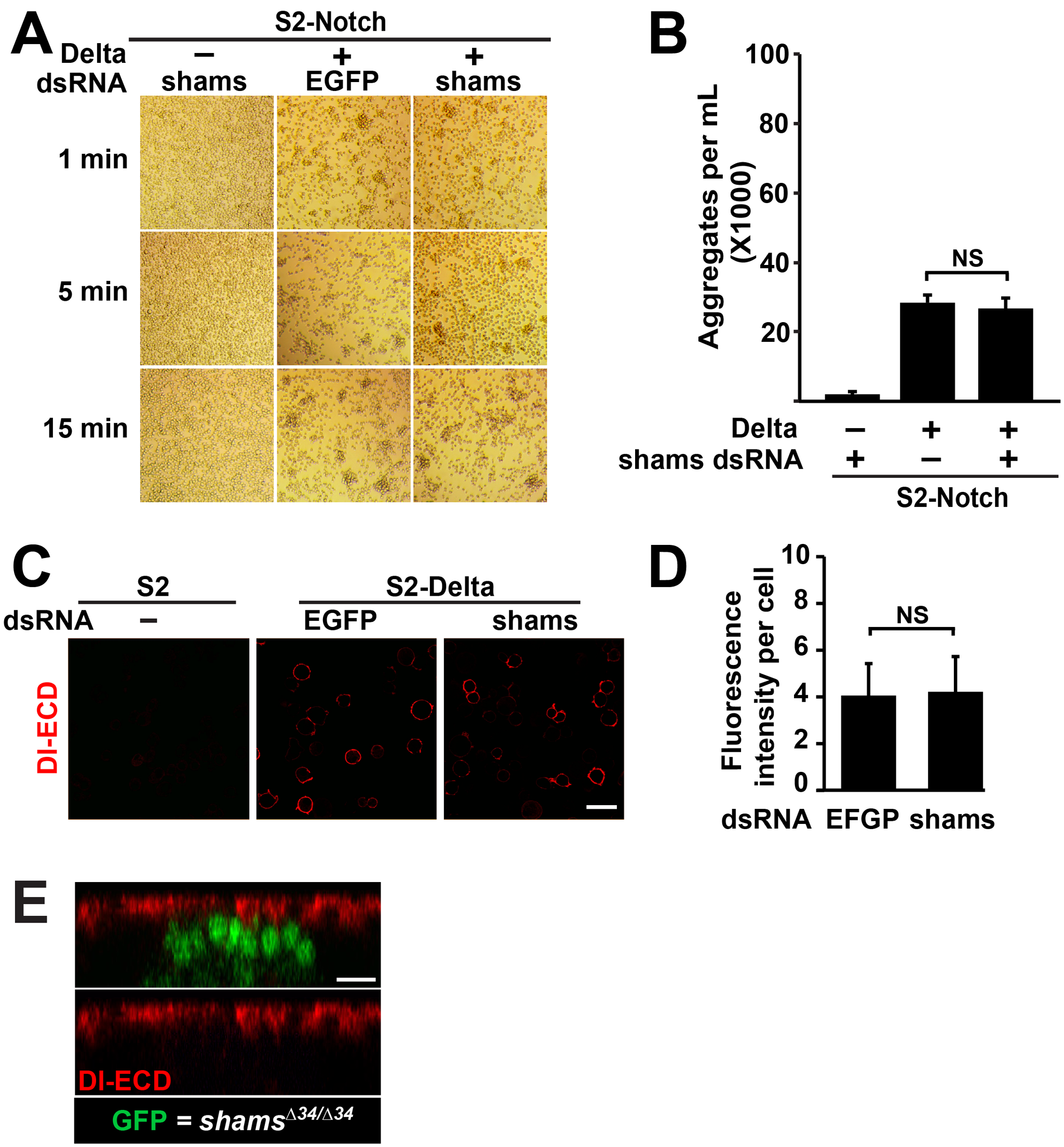

Supplement: S3 Fig — (A) Cell aggregation assays were performed between S2-N cells with S2 cells treated with shams dsRNA (Shams KD), S2-Dl cells treated with a control dsRNA (EGFP) or S2-Dl cells treated with shams dsRNA. Representative images of each co-culture at 1, 5 and 15 minutes are shown. (B) Quantification of number of cell aggregates greater than 6 cells after 5 minutes of co-culture. Error bars indicate standard error. Note that Shams KD does not change the number of aggregates significantly (P>0.05). NS: not significant. (C) Representative images showing cell surface Delta expression (red) in S2 cells (control) and S2-Dl cells (treated with EGFP or shams dsRNA). No difference in expression levels is apparent. Scale bar = 25 μm. (D) Graph shows the average fluorescence intensity of surface Delta in dsRNA (EGFP or shams) treated S2-Dl cells (n = 40 cells for each group). Error bars indicate standard error. NS: not significant. (E) Detergent-free immunostaining for Delta extracellular domain (Dl-ECD) in third instar wing imaginal discs harboring shamsΔ34 MARCM clones (marked with GFP; n = 12). No difference in surface level of Delta between wild-type and mutant cells is apparent. Scale bar = 25 μm. (TIF) [file pgen.1006723.s003.tif]
